# Risk Factors for Hemorrhagic Stroke among Adults in the Democratic Republic of the Congo: A Hospital-Based Study in a Limited Resource Setting

**DOI:** 10.1155/2022/7840921

**Published:** 2022-11-07

**Authors:** Jacques Mbaz Musung, Placide Kambola Kakoma, Marcellin Bugeme, Jeef Paul Munkemena Banze, Clarence Kaut Mukeng, Orly Ngungwa Muyumba, Berthe Mwad Kamalo, Harvey Kabulo Kapya, Ghyslain Lambo Ngongo, Laurent Kitwa, Evariste Tshibind Yav, Olivier Mukuku, Emmanuel Kiyana Muyumba

**Affiliations:** ^1^Department of Internal Medicine, University of Lubumbashi, Lubumbashi, Democratic Republic of the Congo; ^2^Neurology Department, University of Lubumbashi, Lubumbashi, Democratic Republic of the Congo; ^3^Department of Public Health, University of Lubumbashi, Lubumbashi, Democratic Republic of the Congo; ^4^Department of Research, Institut Supérieur des Techniques Médicales de Lubumbashi, Lubumbashi, Democratic Republic of the Congo

## Abstract

**Background:**

The prevalence of stroke is increasing in sub-Saharan Africa. The scarcity of hospital-based stroke data in Lubumbashi (in the Democratic Republic of the Congo) led to the study, which was designed to describe the epidemiology of stroke and identify risk factors associated with hemorrhagic stroke among adult patients in Lubumbashi.

**Methods:**

This was a cross-sectional study of 158 adult patients admitted for stroke in the internal medicine department of Lubumbashi University Clinics from January 2018 to December 2020. Sociodemographic and clinical features, cardiovascular risk factors, and hospital mortality were collected. A logistic regression has determined the risk of developing a hemorrhagic stroke.

**Results:**

Of 9,919 hospitalized patients, 158 had a stroke with a hospital prevalence of 1.6%; 86 (54.4%) patients had a hemorrhagic stroke while 72 (45.6%) had an ischemic stroke. Of which 41.1% (65/158) were women. The mean age was 60.8 ± 13.3 years. Main clinical signs were hemiplegia (63.3%), headache (48.7%), speech disorders (38.6%), and dizziness (38.6%). Hypertension (82.9%) and hyperglycemia (53.2%) were the most common risk factors. Inhospital mortality was 22.8%. After logistic regression, independent predictors for developing hemorrhagic stroke were hypertension (aOR = 8.19; 95% CI: 2.72–24.66; *p* < 0.0001) and atrial fibrillation (aOR = 4.89; 95% CI: 1.41–16.89; *p* = 0.012).

**Conclusion:**

This study highlights the high stroke mortality in a resource-limited hospital and the burden of hypertension in the development of hemorrhagic stroke. It illustrates the need to establish stroke care setting to improve the quality of stroke care.

## 1. Introduction

Stroke is one of the leading causes of morbidity, mortality, and disability worldwide [1, 2]. The majority of these mortality-related strokes occur in developing countries [3]. In sub-Saharan Africa (SSA), hospital epidemiological studies show a lower overall incidence than in developed countries [4]. In the Democratic Republic of the Congo (DRC), the hospital prevalence was 11.7% in Lubumbashi [5]. Risk factors such as age, gender, hypertension, diabetes mellitus, smoking, atrial fibrillation, dyslipidemia, family history of stroke, low income, and low educational level contribute to the increase in stroke prevalence in SSA [6, 7] and make stroke a public health issue in SSA [8]. It has recently been shown that other factors such as metabolic adaptation [9] and genetic factors are involved in the occurrence of stroke [10]. Additionally, among etiological factors, we can list the population's lack of awareness of stroke warning symptoms and poorly-controlled blood pressure (which is partly attributed to the lack of access to hypertensive medications) [11]. In the Congolese study conducted in Kinshasa by Connor et al. [4], hypertension was reported as the commonest identifiable risk factor of stroke in 81%. In Lubumbashi (DRC), a community study reported a high prevalence of hypertension (33.6%) [12], suggesting a high risk of stroke in this population.

It should be recalled here that options for specialized care and diagnosis remain limited or nonexistent for most developing countries [3], including the DRC, where many health facilities are not equipped with computed tomography (CT scan) or MRI. Stroke is associated with increased neuroimaging investigations and surveillance, unplanned or longer stays in hospital and sometimes in intensive care units (ICU), and an increased risk of early rehospitalization. Confirmation of stroke diagnosis depends on neuroimaging, which is expensive (about US$300) and less accessible in our settings.

The lack of available literature on the epidemiology of stroke in our settings had motivated the present study, which aimed to describe the epidemiology of stroke and identify risk factors associated with hemorrhagic stroke among adult patients in Lubumbashi, in the DRC.

## 2. Methods

### 2.1. Study Design and Setting

A retrospective cross-sectional study was conducted on hospitalized stroke patients in the Department of Internal Medicine of Lubumbashi University Clinics from January 2018 to December 2020. It is the main referral center for all diseases in this part of the DRC.

### 2.2. Eligibility Criteria and Sample Size

All patients above 18 years, with confirmed diagnosis of first-in-lifetime or recurrent strokes (evaluated by a neurologist at admission), with complete medical records were included in the study. Hence, a total of 158 stroke patients who had been hospitalized during the three-year (2018–2020) study period were used.

### 2.3. Data Collection

Clinical data were collected using a structured questionnaire which included sociodemographic data (age, gender, educational level, and occupation); risk factors of stroke including hypertension, diabetes mellitus, cardiovascular diseases, hyperlipidemia, smoking, alcohol use, and other less common factors; detailed clinical and neurological examination; electrocardiogram (ECG) results, CT scan results, and laboratory investigations. Rates of hypertension and diabetes mellitus were obtained from the medical history and clinical investigations. Hypertension was diagnosed in patients with systolic blood pressure ≥ 140 mmHg and/or diastolic blood pressure ≥ 90 mmHg, and/or a history of hypertension, reported by a healthcare professional, and/or taking antihypertensive medication before stroke [13]. Diabetes mellitus was diagnosed on the basis of complete overnight fasting plasma glucose concentrations ≥126 mg/dL or random blood glucose of greater than 200 mg/dL and/or a history of diabetes and current treatment with hypoglycemic agents [14]. Hyperlipidemia (code E78.5 of ICD-10) was defined as elevation of serum total cholesterol (≥200 mg/dL) and/or triglycerides (≥150 mg/dL) or reduced high-density lipoprotein cholesterol (<40 mg/dL in men or<50 mg/dL in women) or lipid lowering treatment [15]. Moreover, atrial fibrillation was defined as an abnormal irregular heart rhythm characterized by a fast and irregular heartbeat, absence of P waves, and no pattern to R wave occurrence on ECG.

Brain CT scan was performed routinely for all 158 patients. The distinction between hemorrhagic and ischemic stroke types was based on the CT scan.

### 2.4. Statistical Analysis

Statistical analysis was performed using Stata software version 16.0 for Windows. Data for quantitative variables were presented as the mean with its standard deviation as appropriate and for qualitative variables as counts and percentage. Pearson's chi-squared test was used to compare proportions and Student's *t*-test for comparison of means between two sexes. To estimate the probability of hemorrhagic stroke based on factors related, bivariate analysis was performed followed by multiple logistic regression; *p* value <0.05 was considered statistically significant.

### 2.5. Ethical Considerations

This study was approved by the medical ethical committee of the University of Lubumbashi (Approval number: UNILU/CEM/089/2018). The permission to collect data from medical records was provided by Lubumbashi University Clinics administration. The use of collected data was confidential and anonymous.

## 3. Results

Of a total of 9,919 patients registered during the study period, 158 had a stroke, corresponding to a prevalence of 1.6%. Nearly 59% (93/158) of the patients admitted for stroke were men. The mean age was 60.8 ± 13.3 years. Hemorrhagic stroke was more common (54.4%). Thirty-eight (30.38%) patients had completed tertiary education, and 37 (23.42%) patients were unemployed. We recorded 36 deaths, a hospital mortality of 22.78%. However, death rate was higher in patients with hemorrhagic stroke than in those with ischemic stroke, without statistically significant difference (OR = 0.52 (0.24-1.12); *p* = 0.1369). There was no significant association between demographic characteristics and stroke subtypes (*p* > 0.05) (Table 1).

**Figure 1:**
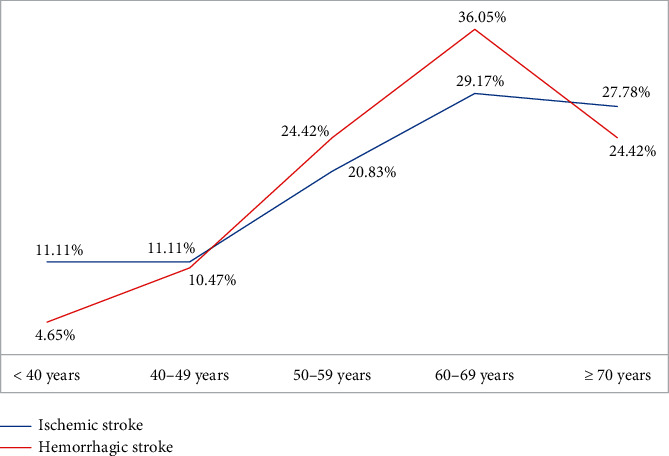
Prevalence of stroke subtypes by age group.

The prevalence of ischemic and hemorrhagic stroke increased with age. This relationship showed an inverted U shape, with the highest levels at 60-69 years. Based on the mixed linear model, the overall association between age group and subtypes of stroke showed a linear increase, with hemorrhagic stroke prevalence exceeding ischemic stroke prevalence from age 40 and becoming lower at age 70 and over (Figure 1).

Main clinical signs were hemiplegia (63.3%), headache (48.7%), speech disorders (38.6%), dizziness (38.6%), fatigue (31.6%), facial paralysis (31.6%), unconsciousness (24%), and seizures (17%) (Table 2). Hypertension (82.9%) and hyperglycemia (53.2%) were the most common risk factors. Analysis of possible relations between comorbid risk factors and stroke subtypes using chi-squared test showed that hypertension (*p* = 0.0012) and atrial fibrillation (*p* = 0.0108) were risk factors statistically significantly associated with hemorrhagic stroke. There was no significant difference between ischemic and hemorrhagic stroke with regard to other risk factors (Table 3).

Table 4 shows the comparison of laboratory investigation values between patients with ischemic and hemorrhagic stroke. No statistical differences were noted when comparing average laboratory investigation values between these subtypes of stroke (*p* > 0.05).

After logistic regression, the risk of hemorrhagic stroke was more than 8-fold in hypertensive patients (adjusted OR = 8.19 (2.72-24.66)) and more than 4-fold in patients with atrial fibrillation (adjusted OR = 4.89 (1.41-16.89)) (Table 5).

## 4. Discussion

The prevalence of stroke in the present study was 1.6%, and the risk factors of hemorrhagic stroke were hypertension and atrial fibrillation. As in the present study, literature data showed that various African studies reported low rates of hospital-based stroke prevalence [16–19], compared to rates reported in developed countries that appear to be high (4.5% in Spain [20] and 7.6% in Germany [21]). Given that our study population is drawn from a proportion of people who performed the CT scan, being completely black (at high risk of high blood pressure compared to other races), we believe that the prevalence reported in this study would be underestimated not reflecting the real situation in our low-resource environment. Also, lack of access to quality care is said to be one of the reasons for low hospital prevalence in low-income regions. Most people do not have health insurance, and medical expenses are their own responsibility. Most stroke patients do not have access to this examination due to lack of financial resources. Given the costs of investigating and managing a stroke, many stroke cases go unseen in hospital and remain undiagnosed.

A previous Congolese study had reported 54.4% hemorrhagic stroke [2], which was higher than 52% reported in the present study. These authors mentioned that this high proportion of hemorrhagic stroke in their study was due to severe and uncontrolled hypertension [2]. Indeed, our recent report on stroke with the diagnosis of stroke subtypes defined by brain CT scan found similarity with other African studies [22–24] finding high rates of hemorrhagic stroke. Our findings are contrary to those reported in other African hospital surveys, which found a high rate of ischemic stroke [16, 17, 25–28].

The present study reports male predominance, but no statistically significant difference as observed in previous studies [17, 25, 29, 30]. Male exposure to cardiovascular risk factors may justify male predominance in stroke. However, the sex-specific disparity in prevalence described in the African literature showed that prevalence was either female [4, 27, 31, 32] or male [17, 18, 29, 30, 33, 34].

The mean age of onset of stroke was 60.89 ± 13.33 years, and the 60-69 age group had the highest prevalence of stroke in this study. This finding was consistent with other surveys in SSA [17, 22, 25, 33–35]. Our results can be superimposed on the study by Coulibaly et al. [16] carried out in Mali, which had a mean age of 62.2 years, and the age group 60 to 75 years was the most affected. In Lubumbashi (in the DRC), Mukeng et al. [5] reported a similar age in a survey conducted in an ICU. The increase in age associated with other cardiovascular risk factors is responsible for the high prevalence of stroke over the age of 60.

Hypertension is described as one of the major risk factors for stroke [17, 35, 36]. Unlike in developed countries, the prevalence of stroke in hypertensive patients is increasing in developing countries [22–24]. In the present study, 82.91% of patients were hypertensive, 94.19% of patients with hemorrhagic stroke, and 69.44% with ischemic stroke. Our results confirm various observations in the literature [22–24, 35, 36] which have shown that hypertension is the most common risk factor in stroke patients in both developing and developed countries. This may reflect a low level of public awareness, the lack of adequate screening, prevention, management, and control of hypertension in our community in particular and in the world in general. In SSA, it was estimated that controlled hypertension would be the major key to significantly reducing the prevalence of stroke since 46% of adults over 25 years of age suffer from hypertension and expose them to a higher risk of developing stroke [37].

The present study showed that hemiplegia was the most common reason for consultation followed by headache. Similar studies have shown similar findings for hemiplegia [38–41]. In contrast to the survey at Jimma University Medical Center in southwestern Ethiopia, Fekadu et al. [26] found headache as the primary reason for consultation followed by aphasia and hemiplegia. Patients with atrial fibrillation were four times more likely to develop hemorrhagic stroke (adjusted OR = 4.89) than ischemic stroke. This finding was consistent with previous African studies had identified atrial fibrillation as the cause of hemorrhagic stroke [26, 42, 43].

The present study found a mortality rate of 22.78%; death rate was higher in patients with hemorrhagic stroke than in those with ischemic stroke, without statistically significant difference (unadjusted OR = 0.52; 95% CI: 0.24-1.12; *p* = 0.1369). This observation was consistent with the literature that has shown that, compared to developed countries, the percentage of hemorrhagic stroke mortality is higher in SSA and other developing countries [44–46].

Stroke is not a fatality when management takes place in well-equipped health facility. However, in the context of resource-constrained countries, the main challenge in the widespread implementation of care in hospital facilities would probably be the cost of basic infrastructure and adequately-qualified healthcare workers. It is true that the system of developed countries can be used to improve stroke prevention and management in developing countries in order to reduce the mortality rate.

As limitations of the study, data collection was retrospective, and some data were not available in some patients' records. The small sample size, which included only the patients hospitalized in internal medicine department and who had performed a brain CT scan, does not reflect the true hospital prevalence of stroke at Lubumbashi University Clinics. Furthermore, the current study was a hospital-based study, and it may be difficult to generalize the findings to the community.

## 5. Conclusion

Clinical and prognostic features of stroke in our settings were similar to those observed in other developing countries. Hypertension and atrial fibrillation were the significant independent predictors of hemorrhagic stroke. The development of a program on risk factor education including hypertension in developing countries is essential to reduce the incidence of stroke.

## Figures and Tables

**Table 1:** Distribution of age, gender, educational level, occupation, and discharge status as well as their association with acute stroke subtypes.

Variable	Total (*n* = 158)	Ischemic stroke (*n* = 72)	Hemorrhagic stroke (*n* = 86)	Unadjusted OR (95% CI)	*p* value
*Age*					
<40 years	12 (7.59%)	8 (11.11%)	4 (4.65%)	1.00	
40-49 years	17 (10.76%)	8 (11.11%)	9 (10.47%)	0.45 (0.07-2.36)	0.4514
50-59 years	36 (22.78%)	15 (20.83%)	21 (24.42%)	0.36 (0.07-1.67)	0.1867
60-69 years	52 (32.91%)	21 (29.17%)	31 (36.05%)	0.34 (0.07-1.48)	0.1190
≥70 years	41 (25.95%)	20 (27.78%)	21 (24.42%)	0.48 (0.09-2.15)	0.3370
Mean ± SD	60.89 ± 13.33	59.67 ± 14.43	61.91 ± 12.33		0.2987

*Gender*					
Female	65 (41.14%)	33 (45.83%)	32 (37.21%)	1.42 (0.75-2.70)	0.3498
Male	93 (58.86%)	39 (54.17%)	54 (62.79%)	1.00	

*Educational level*					0.9246
Primary	43 (27.22%)	19 (26.39%)	24 (27.91%)	1.00	
Secondary	67 (42.41%)	30 (41.67%)	37 (43.02%)	1.02 (0.47-2.21)	1.0000
Higher	48 (30.38%)	23 (31.94%)	25 (29.07%)	1.16 (0.51-2.65)	0.8840

*Occupation*					
Employed	74 (46.84%)	35 (48.61%)	39 (45.35%)	1.00	
Self-employed	47 (29.75%)	21 (29.17%)	26 (30.23%)	0.90 (0.43-1.87)	0.9248
Unemployed	37 (23.42%)	16 (22.22%)	21 (24.42%)	0.85 (0.38-1.88)	0.8399

*Discharge status (hospital outcome)*					
Live	122 (77.22%)	60 (83.33%)	62 (72.09%)	1.00	
Death	36 (22.78%)	12 (16.67%)	24 (27.91%)	0.52 (0.24-1.12)	0.1369

**Table 2:** Prevalence of clinical signs of stroke.

Variable	Total (*n* = 158)	Ischemic stroke (*n* = 72)	Hemorrhagic stroke (*n* = 86)	Unadjusted odds ratio (95% confidence interval)	*p* value
Hemiplegia	100 (63.29%)	45 (62.50%)	55 (63.95%)	0.94 (0.49-1.79)	0.9816
Headache	77 (48.73%)	32 (44.44%)	45 (52.33%)	0.73 (0.39-1.36)	0.4080
Speech disorders	61 (38.61%)	22 (30.56%)	39 (45.35%)	0.53 (0.27-1.02)	0.0821
Dizziness	61 (38.61%)	29 (40.28%)	32 (37.21%)	1.14 (0.60-2.16)	0.8176
Facial paralysis	50 (31.65%)	25 (34.72%)	25 (29.07%)	1.29 (0.66-2.54)	0.5557
Fatigue	50 (31.65%)	27 (37.50%)	23 (26.74%)	1.64 (0.84-3.23)	0.2019
Unconsciousness	38 (24.05%)	15 (20.83%)	23 (26.74%)	0.72 (0.34-1.51)	0.4971
Seizures	28 (17.72%)	8 (11.11%)	20 (23.26%)	0.41 (0.17-1.00)	0.0747

**Table 3:** Prevalence of comorbid risk factors of stroke.

Variable	Total (*n* = 158)	Ischemic stroke (*n* = 72)	Hemorrhagic stroke (*n* = 86)	Unadjusted odds ratio (95% confidence interval)	*p* value
Alcohol consumption	50 (31.65%)	20 (27.78%)	30 (34.88%)	0.72 (0.36-1.41)	0.4326
Cigarette smoking	23 (14.56%)	11 (15.28%)	12 (13.95%)	0.90 (0.37-2.18)	0.9931
Hypertension	131 (82.91%)	50 (69.44%)	81 (94.19%)	0.18 (0.07-0.52)	0.0012
Diabetes mellitus	31 (19.62%)	13 (18.06%)	18 (20.93%)	0.90 (0.37-2.18)	0.9931
Atrial fibrillation	22 (13.92%)	4 (5.56%)	18 (20.93%)	0.22 (0.05-0.73)	0.0108
Hyperglycemia	84 (53.16%)	39 (54.17%)	45 (52.33%)	1.07 (0.57-2.02)	0.9434
Hyperlipidemia	78 (49.37%)	33 (45.83%)	45 (52.33%)	0.77 (0.41-1.44)	0.5136
Hyperuricemia	60 (37.97%)	26 (36.11%)	34 (39.53%)	0.86 (0.43-1.73)	0.7817
Acute kidney injury	87 (55.06%)	39 (54.17%)	48 (55.81%)	0.93 (0.50-1.75)	0.9627

**Table 4:** Comparison of mean values of laboratory investigation data of patients with ischemic and hemorrhagic stroke.

Variable	Total (*n* = 158)	Ischemic stroke (*n* = 72)	Hemorrhagic stroke (*n* = 86)	*p* value^∗^
Creatinine (mg/dL), mean ± SD	2.11 ± 1.68	2.02 ± 1.50	2.19 ± 1.82	0.533
Urea (mg/dL), mean ± SD	67.76 ± 48.34	65.39 ± 48.04	69.78 ± 48.81	0.586
Fasting blood sugar (mg/dL), mean ± SD	163.06 ± 104.05	149.07 ± 76.35	175.10 ± 122.23	0.131
Uric acid (mg/dL), mean ± SD	5.86 ± 2.62	5.68 ± 2.58	6.01 ± 2.66	0.435
Total cholesterol (mg/dL), mean ± SD	193.25 ± 50.20	194.11 ± 49.93	192.49 ± 50.74	0.842
LDL cholesterol (mg/dL), mean ± SD	116.39 ± 40.92	116.53 ± 39.12	116.28 ± 42.91	0.969
Triglycerides (mg/dL), mean ± SD	139.34 ± 55.97	137.91 ± 56.85	140.58 ± 55.52	0.771
HDL cholesterol (mg/dL), mean ± SD	62.80 ± 16.38	61.99 ± 17.53	63.52 ± 15.36	0.566

^∗^ Student's *t*-test. HDL: high-density lipoprotein; LDL: low density lipoprotein; SD: standard deviation.

**Table 5:** Logistic regression analysis of risk factor of hemorrhagic stroke.

Variables	Adjusted odds ratio (95% confidence interval)	*p* value
Hypertension	8.19 (2.72-24.66)	<0.0001
Atrial fibrillation	4.89 (1.41-16.89)	0.012
Diabetes mellitus	1.42 (0.58-3.49)	0.439
Hyperlipidemia	0.99 (0.49-1.99)	0.987

## Data Availability

The datasheet used to support the findings of this study is available from the corresponding author upon request.

## Abbreviations

95% CI: 95% confidence interval
aOR: Adjusted odds ratio
CT: Computed tomography
DRC: Democratic Republic of the Congo
ECG: Electrocardiogram
ICU: Intensive care unit
SSA: Sub-Saharan Africa.
